# Analysis of Metabolomic Reprogramming Induced by Infection with Kaposi’s Sarcoma-Associated Herpesvirus Using Untargeted Metabolomic Profiling

**DOI:** 10.3390/ijms26073109

**Published:** 2025-03-28

**Authors:** Abdulkarim Alfaez, Michael W. Christopher, Timothy J. Garrett, Bernadett Papp

**Affiliations:** 1 Department of Pathology, Immunology, and Laboratory Medicine, University of Florida, College of Medicine, Gainesville, FL 32610, USA; krmmohmmad@ufl.edu; 2 Department of Clinical Laboratory Sciences, College of Applied Medical Sciences, King Saud University, Riyadh, 11433, Saudi Arabia; 3 Department of Chemistry, University of Florida, Gainesville, FL 32611, USA; m.christopher@ufl.edu; 4 Department of Oral Biology, University of Florida College of Dentistry, Gainesville, FL 32610, USA; 5 Genetics Institute, University of Florida, Gainesville, FL 32610, USA; 6 Health Cancer Center, University of Florida, Gainesville, FL 32610, USA; 7 Informatics Institute, University of Florida, Gainesville, FL 32610, USA; 8 Center for Orphaned Autoimmune Disorders, University of Florida, Gainesville, FL 32610, USA

**Keywords:** metabolomics, KSHV, de novo infection, oral epithelial cells

## Abstract

Kaposi’s sarcoma-associated herpesvirus (KSHV) is an oncogenic double-stranded DNA virus. There are no vaccines or antiviral therapies for KSHV. Identifying the cellular metabolic pathways that KSHV manipulates can broaden the knowledge of how these pathways contribute to sustaining lytic infection, which can be targeted in future therapies to prevent viral spread. In this study, we performed an untargeted metabolomic analysis of KSHV infected telomerase-immortalized gingival keratinocytes (TIGK) cells at 4 h post-infection compared to mock-infected cells. We found that the metabolomic landscape of KSHV-infected TIGK differed from that of the mock. Specifically, a total of 804 differential metabolic features were detected in the two groups, with 741 metabolites that were significantly upregulated, and 63 that were significantly downregulated in KSHV-infected TIGK cells. The differential metabolites included ornithine, arginine, putrescine, dimethylarginine, orotate, glutamate, and glutamine, and were associated with pathways, such as the urea cycle, polyamine synthesis, dimethylarginine synthesis, and de novo pyrimidine synthesis. Overall, our untargeted metabolomics analysis revealed that KSHV infection results in marked rapid alterations in the metabolic profile of the oral epithelial cells. We envision that a subset of these rapid metabolic changes might result in altered cellular functions that can promote viral lytic replication and transmission in the oral cavity.

## 1. Introduction

Metabolomics is the analysis of a vast set of metabolites that provides insight into the physiological aspect of cellular metabolism [1]. Metabolites and substrates are essential components of a cellular process that influence cellular functions. Metabolomic profiling is performed using mass spectrometry (MS) approaches, typically with the combination of liquid chromatography-high-resolution mass spectrometry (LC-HRMS). LC-HRMS is widely used in the detection of metabolites because of its ability to analyze a broad spectrum of molecules with high sensitivity and specificity. Targeted and non-targeted approaches are applied to detect known and unknown metabolites, respectively, to investigate the underlying mechanisms of a specific pathway [2,3]. Metabolomics is crucial to fully understand a pathophysiological state. Homeostatic mechanisms could be evaluated under different conditions, including their normal state and after encountering environmental stimuli, including viruses.

Kaposi’s sarcoma-associated herpesvirus (KSHV), also known as Human herpesvirus 8, is an oncogenic pathogen. KSHV is a gammaherpesvirus with a large double-stranded DNA genome that can cause various cancers and lymphoproliferative disorders [4,5]. Like all herpesviruses, KSHV has a biphasic life cycle, latent and lytic life cycles, and is highly regulated by cellular signaling pathways upon entry in the cells [6,7,8]. In the primary infection, KSHV deposits its DNA, which is gradually heterochromatinized to establish latency in most cell types. During latency, latent genes are expressed and latent viral factors maintain a lifelong persistence by evading immune surveillance, promoting cell survival and proliferation, and promoting maintenance of the KSHV genome through cell divisions [9,10]. However, various stimuli, such as secondary infection, can induce the virus lytic genes in a sequential manner, starting with immediate early genes (IE), then early (E), and finally late genes (L).

Viruses, including KSHV, depend on the host’s cellular machinery to support viral replication and survival since they cannot synthesize their metabolites. This is considered one of the obstacles to viral propagation and pathogenesis. KSHV has several approaches to hijack the cellular metabolism to support its production and pathogenesis throughout its life cycle [11]. KSHV can impact the cellular metabolism to promote host cell survival and proliferation. The metabolic indication of KSHV-infected cells has a similar characteristic to cancer cells; thus, similarities exist between cancer cells and KSHV-infected cells [12,13,14]. Oncogenic viruses such as KSHV support their pathogenesis by manipulating major cellular host metabolisms such as the central carbon metabolism, highlighting the metabolic pathways’ crucial role in regulating the outcome of virus infection and its effect on host cells [15]. The KSHV infection of cells can activate aerobic glycolysis, leading to lactic acid production. This effect matches the Warburg effect in cancer cells by increasing glucose consumption and lactate production at a normal oxygen level [16]. In addition, KSHV-encoded lytic factors (such as K5) can promote oncogenesis in endothelial cells by increasing aerobic glycolysis and lactate production [17]. Hijacking the host metabolism could also promote viral infection by evading the innate immune system. Metabolites and metabolic signaling can regulate the innate immunity against viruses by manipulating the innate regulatory MAVS [18]. For instance, excessive lactate production from the glycolytic pathway impairs the aggregation of MAVS. The MAVS protein is required to trigger Type 1 interferon response as a defense mechanism against viruses [19]. Furthermore, it has been shown that KSHV-infected cells, through latent proteins (including LANA), can upregulate enzymes in the glutamine pathways, suggesting that KSHV interferes with the host glutaminolysis to induce KSHV-transformed cell proliferation [20]. Glutamine is considered a significant amino acid that generates energy. Additionally, fatty acids are essential in the synthesis of all biological membranes and for the energy production of cells. Importantly, KSHV activates and subsequently relies on metabolic pathways, including the fatty acid synthesis pathway, for the survival of the latently infected cells as well as for its lytic replication and virion production [14].

KSHV can enter the body through the oral cavity. The oral epithelium provides an environment for KSHV replication that leads to the infection of other cells, particularly tonsillar B cells, to establish a persistent infection [21,22,23]. The detection of KSHV DNA in the saliva suggests that it is the primary mode of KSHV transmission [23,24,25,26,27]. KSHV can infect oral epithelial cells in which KSHV euchromatin and lytic gene expression is sustained, and this can result in lytic viral replication [10,25,28]. We recently demonstrated that the infection of oral epithelial cells by KSHV rapidly alters the expression of host genes, which contributes to the sustained lytic infection [28].

Metabolic reprogramming events are also known to influence the viral life cycle in a highly dynamic manner; however, they remain less characterized [13]. The identification of altered metabolites can help to uncover their potential role in supporting KSHV primary lytic infection; then, a metabolic pathway could be targeted to prevent lytic infection. Here, we aimed to identify the metabolic reprogramming events in oral epithelial cells during de novo KSHV infection of oral epithelial cells at four hours post-infection (hpi). To address this question, we performed liquid chromatography–high-resolution/mass spectrometry (LC-HRMS) to separate and detect the metabolites in KSHV-infected TIGK cells and compare them to uninfected (mock) TIGK cells. Our analysis revealed that KSHV infection alters the global metabolomic landscape of TIGK cells. Future investigations can determine if our newly identified rapid metabolomic reprogramming events during the first hours of KSHV infection can support sustained primary lytic infection in oral epithelial cells.

## 2. Results

### 2.1. Untargeted Metabolomics Analysis of De Novo KSHV Infection in Oral Epithelial Cells

**Figure 1:**
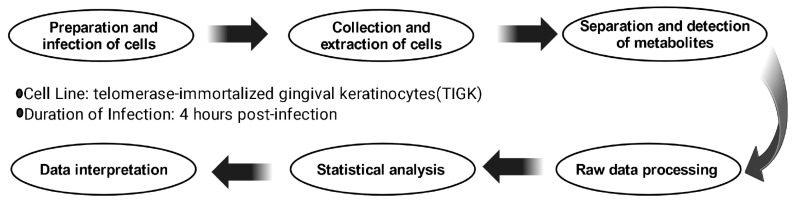
Schematic summary of the applied untargeted metabolomics workflow.

Our goal was to reveal the alterations in the metabolic profile of telomerase-immortalized gingival keratinocytes (TIGK) at 4 h following KSHV infection compared to uninfected control TIGK cells (mock). To this end, an untargeted metabolomic study was performed. As summarized on Figure 1, three biological replicates of KSHV-infected cells and mock-infected cells were collected, metabolites were extracted and analyzed in both positive and negative ion modes, and the data were processed via statistical analyses (Figure 1). To characterize the metabolomic profiles of KSHV-infected cells compared to the mock cells, the samples were measured using LC-HRMS in positive and negative ion modes. The dataset of the refined metabolomics comprised a total of 11059 features, comprising 221 identified metabolites (Table S1). From the detected features, we identified 1323 that had a 2-fold change or greater difference between groups (Table S2), and total of 850 features that showed statistically significant differences in expression (*p* value < 0.05 following false discovery rate (FDR) correction) (Table S3), when KSHV-infected cells were compared to the mock.

### 2.2. Identification of Upregulated or Downregulated Metabolites upon KSHV Infection

**Figure 2:**
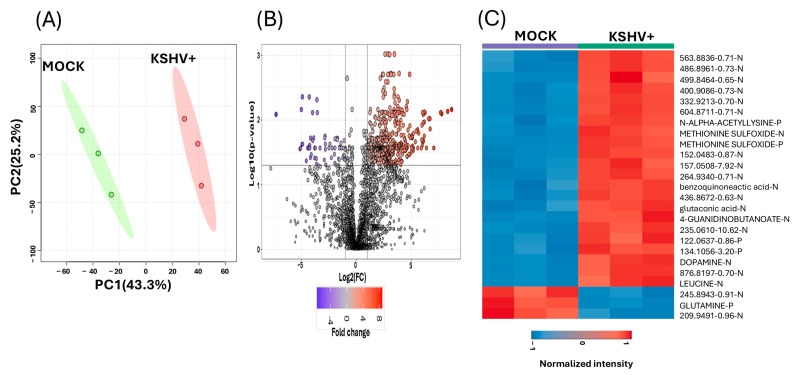
Metabolites profile of KSHV-infected cells and mock cells. (**A**) Two-dimensional principal component analysis score plot compares the metabolomic profile of KSHV-infected cells to mock cells. In the plot, green represents mock cells and red represents KSHV-infected cells. (**B**) Volcano plot illustrates the metabolomic profile of KSHV-infected cells compared to mock cells. In this plot, the red circle represents the significantly upregulated metabolites in KSHV-infected cells, and the purple circle represents the significantly downregulated metabolites. (**C**) Hierarchical clustering heat map displays the metabolic abundance in KSHV-infected cells compared to mock cells. Red color represents the significantly upregulated metabolites, and the blue color represents the significantly downregulated metabolites. All analyses were conducted for metabolites with a fold change ≥ 2 and a *p* value < 0.05 (FDR). Both Level 1 and Level 3 metabolites were utilized to generate Figure 2. In (**C**), Level 1 is indicated with uppercase letters, while Level 3 is indicated with lowercase letters.

Our analysis consisted of features identified as a specific metabolite with level 1 confidence and others identified by mass-to-charge (*m*/*z*) and retention time that are yet to be identified. Principal components analysis (PCA) was applied to examine the overall metabolome profile between the KSHV-infected and mock-infected cells. Using PCA allows for the preliminary detection of clusters and their distribution. PCA showed separation in the metabolomic profile between KSHV-infected cells and mock-infected cells. PCA indicated that PC1 explained 43.3% of the variability, while PC2 explained 25.2%, for a total variance of 68.1% (Figure 2A). A volcano plot analysis was performed to determine features with a fold change of 2 or greater and a *p*-value (FDR) of 0.05 or lower. A total of 741 features were significantly upregulated, while 63 were significantly downregulated, when comparing KSHV-infected with mock-infected cells, per the fold change and *p*-value cutoff criteria (Table S4) (Figure 2B). Furthermore, a heatmap was applied to illustrate the abundance of the top 25 significant features. Among the most significantly upregulated metabolites were dopamine, leucine, N-alpha-acetyllysine, gluconic acid, methionine sulfoxide, and benzoquinonacetic acid, while glutamine was downregulated in KSHV-infected cells (Figure 2C).

### 2.3. Discovering Known Metabolites Altered upon KSHV Infection

**Figure 3:**
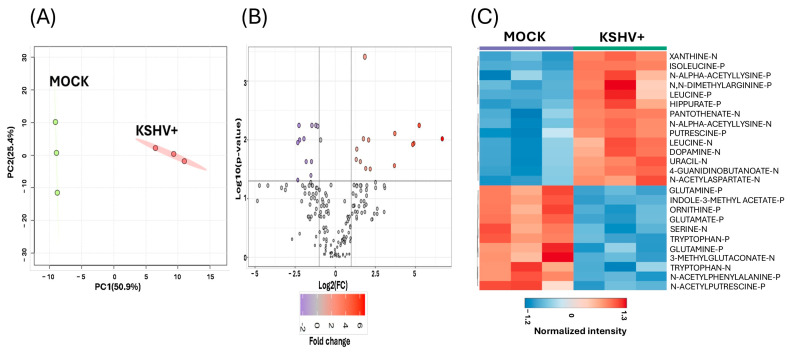
Profile of KSHV infected cell and mock cells showing only identified metabolites. (**A**) 2D-principal component analysis score plot compares the metabolomic profile of KSHV-infected cells to mock cells. In the plot, green represents mock cells and red represents KSHV-infected cells. (**B**) A volcano plot illustrates the metabolomic profile of KSHV-infected cells compared to mock cells. The red circle represents significantly upregulated metabolites in KSHV-infected cells, and the purple circle represents significantly downregulated metabolites. (**C**) The hierarchical clustering heat map displays the metabolic abundance in KSHV-infected cells compared to mock cells. In this heat map, the red color represents significantly upregulated metabolites, and the blue color represents significantly downregulated metabolites All analyses were conducted for metabolites with a fold change ≥ 2 and a *p* value < 0.05 (FDR). Only level 1 metabolites were utilized to generate (**A**–**C**).

The next analysis of the dataset comprised only identified features of a specific metabolite. PCA was conducted on only the known metabolites. PCA showed that the PC1 explained 50.9% of the variability, while PC2 explained 25.4%, indicating the separation of the identified metabolite profile between KSHV-infected cells and mock cells (Figure 3A). Volcano plot analysis was used to identify detected features with a fold change of 2 or greater and a *p*-value (FDR) of 0.05 or lower. We identified 14 that were significantly upregulated, while 12 were significantly downregulated, when comparing KSHV-infected with mock cells (Table S5) (Figure 3B). The heatmap highlights the top 25 identified features (Figure 3C). Among the most significantly upregulated metabolites were dopamine, isoleucine, leucine (positive and negative ion), N-alpha-acetyl lysine, xanthine, dimethylarginine, and uracil, while glutamine, ornithine, serine, tryptophan (positive and negative ions), and aspartate were downregulated in KSHV-infected cells (Figure 3C).

**Figure 4:**
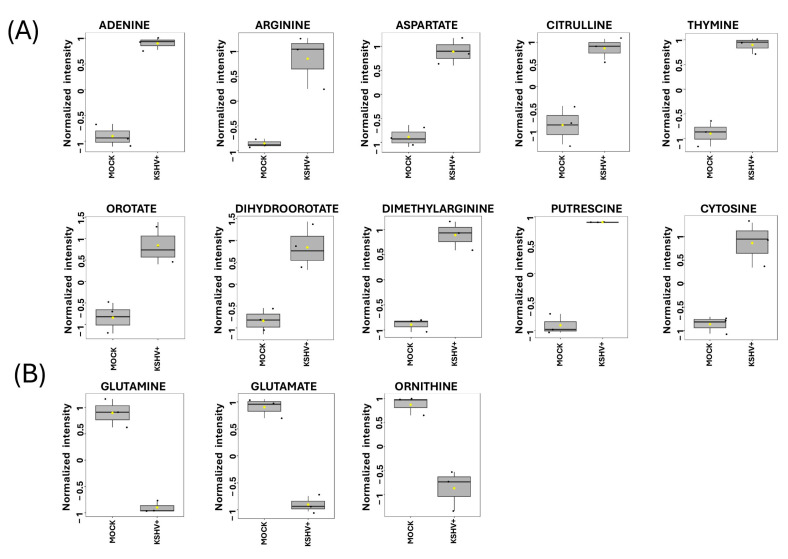
Significant metabolites of KSHV-infected cells. Box plots illustrate the relative signal intensity levels of discriminant metabolites between KSHV-infected cells and mock cells. Metabolites that are significantly upregulated (**A**) or downregulated (**B**) in KSHV-infected cells. The black dots within the plot denote the metabolite levels in the three biological replicates, while the yellow diamond signifies the average value for the group, as denoted on the *y*-axis. This figure displays level 1 metabolites.

Specifically, plots indicate the normalized intensity of significantly altered metabolites in KSHV-infected cells compared to mock cells (Figure 4). Ten of these metabolites were upregulated in KSHV-infected cells compared to mock cells, including adenine (*p <* 0.009), arginine (*p <* 0.03), aspartate (*p <* 0.02), citrulline (*p <* 0.02), dihydroorotate (*p <* 0.04), orotate (*p <* 0.04), dimethylarginine (*p <* 0.01), thymine (*p <* 0.01), putrescine (*p <* 0.006), and cytosine (*p <* 0.02) (Figure 4A). However, three were downregulated, including glutamine (*p <* 0.009), glutamate (*p <* 0.005), and ornithine (*p <* 0.01) (Figure 4B). We envision that these altered metabolites might be involved in pathways that could potentially support KSHV lytic infection in oral epithelial cells. These pathways include the urea cycle, the asymmetrical dimethylarginine (ADMA) and symmetrical dimethylarginine (SDMA) pathways, and de novo pyrimidine biosynthesis.

### 2.4. Enrichment Analysis of the Significantly Altered Metabolites in KSHV-Infected Cells

**Figure 5:**
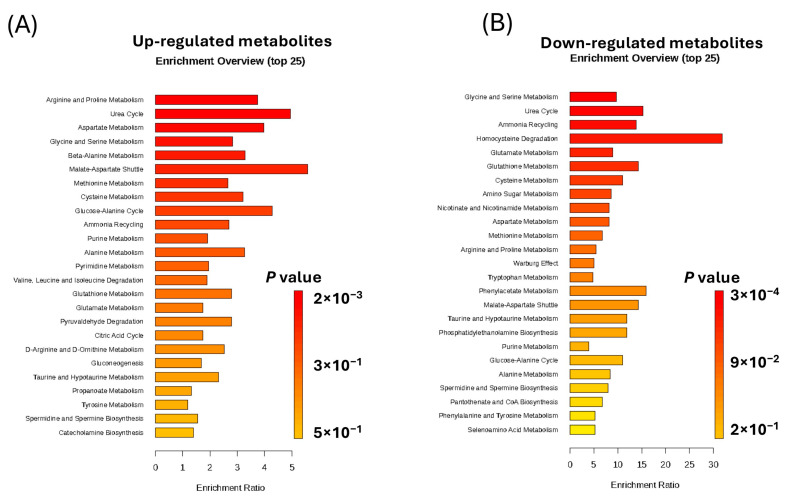
Metabolic enrichment analysis. The enrichment analysis was conducted for the significantly upregulated metabolites in KSHV-infected cells (**A**) and the significantly downregulated metabolites in KSHV-infected cells (**B**) using the Small Molecules Pathway Database (SMPD). The analysis was performed for metabolites with a fold change ≥ 2 and a *p* value < 0.05 (FDR)**.** These figures were generated with both level 1 and 3 metabolites.

**Figure 6:**
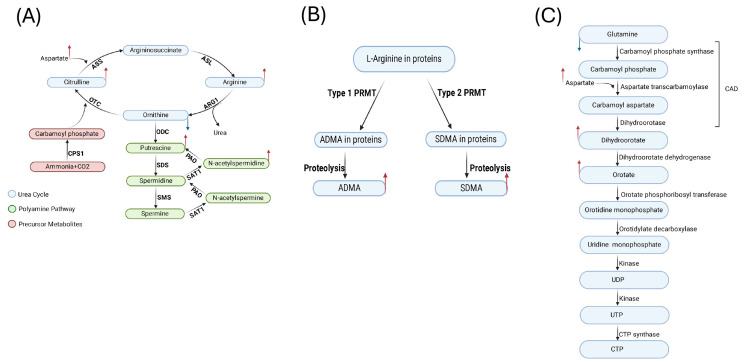
Schematic overview of selected affected pathways in KSHV-infected cells. (**A**) Urea cycle and polyamine pathways; (**B**) ADMA and SDMA pathway; and (**C**) de novo pyrimidine synthesis. The metabolites highlighted with a red arrow indicate upregulated metabolites, while those highlighted with a blue arrow indicate downregulated metabolites. This figure displays level 1 metabolites.

To delve into the pathways associated with metabolic changes, an enrichment analysis was performed on significantly upregulated and downregulated metabolites in KSHV-infected compared to mock cells, respectively (Tables S6 and S7). The outcome of the enrichment analysis of upregulated metabolites with top 25 pathways is shown (Figure 5). Focusing on the upregulated metabolites first, only six metabolic pathways were significantly enriched (raw *p* value *<* 0.05) as follows: arginine and proline metabolism (*p <* 0.01), urea cycle (*p <* 0.002), aspartate metabolism (*p <* 0.006), glycine and serine metabolism (*p <* 0.01), beta-alanine metabolism (*p <* 0.03), and malate–aspartate shuttle (*p <* 0.04) (Figure 5A). Additionally, the enrichment analysis of downregulated metabolites revealed the top 25 downregulated metabolic pathways. Four metabolic pathways were significantly enriched (with FDR *p* value *<* 0.05), as follows: glycine and serine metabolism (*p <* 0.02), urea cycle (*p <* 0.02), ammonia recycling (*p <* 0.02), and homocysteine degradation (*p <* 0.03). In addition, eight metabolic pathways were significantly enriched with (raw *p* value *<* 0.05), as follows: glutamate metabolism (*p <* 0.003), glutathione metabolism (*p <* 0.007), cysteine metabolism (*p <* 0.01), amino sugar metabolism (*p <* 0.01), nicotinate and nicotinamide metabolism (*p <* 0.02), aspartate metabolism (*p <* 0.02), methionine metabolism (*p <* 0.03), and arginine and proline metabolism (*p <* 0.4) (Figure 5B). In summary, our untargeted metabolomics analysis revealed the altered metabolites and their associated pathways in KSHV-infected oral epithelial cells (Figure 6). Future studies are warranted to find out how these altered metabolites and pathways may contribute to lytic de novo KSHV infection.

## 3. Discussion

Multiple studies have been conducted on the metabolomics of KSHV-infected cells, but most studies either focus on already-infected cells or dissect events occurring at a later timepoint, on days following the de novo infection of cells [11,12,14,16,29]. To complement these studies, here we revealed the metabolic changes in oral epithelial cells during the first four hours of primary infection by KSHV. We applied LC-HRMS, which is an unbiased technique for examining the differences in the metabolic profile among biological samples. Overall, our results demonstrate that the KSHV-infected cells have a markedly altered metabolic profile at 4 h compared to control (mock) oral epithelial cells, revealing that primary KSHV infection can induce a rapid change in the metabolic profile of oral epithelial cells. Our findings indicate several metabolites were significantly altered in KSHV-infected cells, such as ornithine, arginine, putrescine, dimethylarginine, orotate, glutamate, and glutamine. These metabolites are part of different pathways, including urea cycle, polyamine synthesis, dimethylarginine synthesis, and de novo pyrimidine synthesis (as summarized in Figure 6).

Importantly, KSHV infection has been described as upregulating the intracellular proline concentration in 3D culture by hijacking Pyrroline-5-carboxylate reductase via K1 oncoprotein, resulting in increased tumor growth [30]. Our study revealed that several amino acids, including arginine, isoleucine, leucine, tryptophan, serine, proline, glutamine, and glutamate, were rapidly dysregulated in oral epithelial cells during KSHV infection. This included several amino acid pathways and free amino acids, possibly synthesized from citric acid cycle intermediates. The free amino acid can be recycled to synthesize glucose and fatty acid and further utilized to produce ATP [31]. It has also been demonstrated that the hypoxic environment is associated with increased KSHV lytic reactivation and generally resulted in downregulated amino acid metabolism genes (including proline, leucine, and arginine metabolism genes) in the KSHV-positive B-cell BJAB cell line due to metabolic reprogramming [32]. Furthermore, arginine, isoleucine, and leucine, which we identified among the upregulated amino acids in KSHV infection, can regulate mTORC1, which controls cell growth and metabolism by binding to the amino acid sensors [33]. KSHV infection might rapidly upregulate the synthesis of amino acids as well as inducing protein degradation to support viral replication in oral epithelial cells.

Notably, we also detected downregulated metabolites in KSHV-infected oral epithelial cells, including tryptophan, serine, glutamine, and glutamate. The degradation of tryptophan metabolite supplements two different pathways, serotonin, and kynurenine. The majority of tryptophan is metabolized within the kynurenine pathway, ultimately resulting in the synthesis of niacin [34], which serves as a precursor for nicotinamide adenine dinucleotide (NAD) molecules. NAD plays a critical role in cellular processes, including cellular respiration, electron transport, redox status, and anabolic metabolism. As an example, NAD acts as a substrate for mono (ADP-ribosyl) transferases and poly(ADP-ribosyl)transferases. These enzymes facilitate the attachment of an adenosine diphosphate ribose (ADP-ribose) moiety to proteins. In cancer cell lines, the post-translational modification mono ADP-ribosylation of histone 3 at arginine 117 (H3R117) has been shown to induce cell proliferation, migration, and colonization [35]. Therefore, we speculate that KSHV may be able to hijack indoleamine 2,3-dioxygenase (IDO or IDO1), which is a rate-limiting enzyme in the Kynurenine pathway, to promote KSHV pathogenesis. Further studies are warranted to investigate the possible impacts of the regulation of these metabolites and their mechanisms.

Our pathway enrichment analysis also revealed altered urea cycle (UC) metabolites in the KSHV-infected cells. The urea cycle metabolites that were dysregulated through KSHV infection include citrulline, arginine, ornithine, and other metabolites that contribute indirectly to the urea cycle, such as glutamate, glutamine, and aspartate. In addition to the primary function of urea cycle enzymes of eliminating the toxic ammonia, it provides its intermediates as a source of endogenous amino acids. In addition, UC enzymes play a vital role in synthesizing and supplementing other metabolites, including polyamine, nitric oxide (NO), and proline, which are critical for cell proliferation and survival [36]. Ammonia is recycled due to the high demand for amino acid synthesis in tumors. The higher demand for nitrogen in tumors drives UC dysregulation in several cancers [37]. Privatt et al. [38] reported that urea concentration was low in plasma from KSHV+ HIV+ patients compared to KSHV+ HIV− patients, revealing the dysregulation of metabolic pathways association with viral co-infection and KS disease states [38].

Furthermore, we showed that metabolite putrescine was higher in KSHV-infected cells compared to mock. Putrescine is part of the polyamine pathway. It is known that viruses package polyamine within their virion to support cellular function for optimal virus replication [39]. Polyamine enhances the activity of viral DNA-dependent DNA polymerase and DNA-dependent RNA-polymerase, including an enzyme encoded by Human herpes virus 1 (HSV1) [39,40]. It is known that KSHV dysregulates the polyamine pathway, specifically affecting the intermediate spermidine and its role in cellular growth and post-translational modification, including hypusination. It was found that the hypusination of eIF5A induces the expression of LANA, a latent KSHV protein, to sustain the KSHV episome by facilitating the translation of polyproline stretches [41]. Notably, a previous study showed that the lytic reactivation of KSHV results in a decrease in intracellular polyamine metabolites [42]. The difference between our findings might be explained by the duration of infection and the utilized cell types. In addition, it was also shown that an increase in the hypusination of eIF5A is necessary for the translation of a major KSHV lytic gene, RTA. Additionally, multiple KSHV viral proteins demonstrated a high frequency of motifs that are dependent on hypusinated eIF5A. Thus, the polyamine pathway is crucial for the translation of KSHV lytic proteins, which facilitates the propagation of the virus [42]. Li et al. [43] reported that P53 induces ammonia accumulation to control tumor growth via inhibiting the urea cycle. The accumulation of ammonia reduces the translation of the ornithine decarboxylase (ODC), a rate-limiting enzyme, in the polyamine biosynthesis pathway [43]. Fiches et al. [42] observed that although the expression of the ODC gene was upregulated, there was no significant change in the intracellular level of polyamines in latent KSHV infection. However, in lytic reactivation, there was a reduction in the intracellular polyamine level [42]. In contrast, our findings suggest that KSHV rapidly dysregulates the urea cycle intermediates and putrescine in the oral epithelial cells during infection. The decrease in ornithine and increase in arginine and putrescine suggest that KSHV may hijack the ODC enzyme in oral epithelial cells. This observation suggests that these two pathways might have a role in lytic infection, which remains to be investigated.

Our data revealed that the dimethylarginine was upregulated in the KSHV-infected cells. Dimethylarginine, including asymmetrical dimethylarginine (ADMA) and symmetrical dimethylarginine (SDMA), is a non-proteogenic amino acid that forms through a post-translation modification of arginine residues that are part of a protein entity [44,45]. The methylation reaction is catalyzed by an enzyme family known as protein arginine methyltransferases (PRMTs) [46,47,48]. A study has shown that the ADMA level is increased in the plasma of HIV patients with/without AIDS [49]. Also, it has been shown that a higher level of ADMA in serum in COVID-19 patients could provide an indicator of the severity of the disease [50]. Higher SDMA and ADMA concentrations were found to be connected with a higher mortality rate in COVID-19 patients [51]. The dysregulation of PRMTs, including Type 1 and 2, is also linked with multiple cancers via histone and non-histone targets [52]. In addition, KSHV vCyclin, a latent KSHV protein, is methylated by PRMT5, which positively regulates its activity and enhances the proliferation and progression of the cell cycle [53]. In contrast, it is known that the overexpression of ORF59, a lytic viral protein, led to a decrease in PRMT5 by reducing the methylation of H4R3, which is known as a repression mark [54]. Thus, KSHV might alter the type 1 and 2 PRMTs, thereby controlling the modifications of specific host and viral proteins during infection.

Our metabolic and enrichment analyses revealed the dysregulation of several de novo pyrimidine synthesis metabolites in KSHV-infected cells. Specifically, upregulated metabolites included dihydroorotate, orotate, cytosine, thymine, and uracil, while only glutamine was downregulated. Pyrimidine provides the nitrogenous base as a DNA and RNA synthesis building block [55]. The pyrimidine nitrogenous bases can be synthesized through either the salvage or de novo pathways. Proliferative cells tend to rely on the de novo synthesis of pyrimidines [56]. Qin et al. [57] reported that SARS-CoV-2 induced de novo pyrimidine synthesis by hijacking carbamoyl–phosphate synthetase, aspartate transcarbamoylase, and dihydroorotase (CAD) enzymes, which provides a rate-limiting step in the de novo pyrimidine synthesis pathway. Viruses manipulate the de novo pyrimidines enzyme to support viral replication and evade inflammatory immune response [57]. CAD induces aerobic glycolysis by deaminating the RelA, an NF-κB subunit, eliminating the inflammatory response of NF-κB-responsive genes [58]. In the context of KSHV infection, a recent elegant study by Wan et al. [29] reported that KSHV reprogrammed the host metabolism to induce the de novo pyrimidine synthesis and aerobic glycolysis by hijacking the CAD enzyme in KSHV-infected cells. In addition, the deamination of ReIA was induced during KSHV de novo infection, which promotes the expression of the glycolytic enzymes pathway [29]. Similarly, our untargeted metabolomics study showed that the intermediates of the pyrimidine pathway were upregulated at 4hpi during KSHV infection, suggesting that the related enzymes and metabolites might be rapidly dysregulated upon KSHV infection and could play an early role in promoting KSHV lytic replication and evading the antiviral immune response. The ultimate goal is to identify how KSHV utilizes the host metabolism to enhance its replication and pathogenesis. The integration of metabolomics with other omics, including genomic, transcriptome, and proteome, is vital to identify and connect biological events during KSHV infection. We utilized unbiased metabolomics and identified specific changes that occur rapidly during KSHV infection in oral epithelial cells, which warrants further investigation. We envision that a subset of our identified altered processes may promote the replication and transmission of KSHV.

## 4. Materials and Methods

### 4.1. Cell Culture and Viral Infection

Telomerase-immortalized gingival keratinocyte (TIGK) cells were obtained from ATCC (Manassas, VA, USA) and cultured in the dermal basal cell medium (ATCC, Manassas, VA, USA), supplemented with keratinocyte growth factors (ATCC, Manassas, VA, USA) following the manufacturer protocols. Growth medium was supplemented with penicillin–streptomycin antibiotics for infection. TIGK cells were spin-infected with KSHV for 45 min. After a PBS rinse, cells were collected at 4 h post-infection using a cell scraper following quenching with iced methanol. Cells were frozen until metabolite extraction.

### 4.2. Metabolite Extraction

Samples were ice-cold throughout the metabolite extraction process to minimize the degradation of metabolites. A protein precipitation procedure was used to extract the metabolites. The cells were first washed with 40 mM ammonium formate, followed by homogenization using a bead beater (Fisher Scientific, Pittsburgh, PA, USA) and the addition ammonium acetate and 0.7 mm zirconia beads. Subsequently, the protein concentration of the samples was determined and the samples were normalized to 5 mg/mL of protein prior to extracting the metabolites using a bicinchoninic acid assay kit (Thermo Scientific (Rockford, IL, USA)). An extraction blank was added and treated similarly to the biological samples for blank feature filtering. An internal standard mixture was added to each sample. The internal standard mix included L-leucine-D10, caffeine-(1-methyl-D3), succinic acid-2,3,3,3-D4 were obtained from CDN Isotopes (Pointe-Claire, QC, Canada), and L-tyrosine-13C6, L-leucine-13C6, and L-phenylalanine-13C6 were obtained from Cambridge Isotope Laboratory (CIL, Tewksbury, MA, USA). L-, N-BOC-L-tert-leucine, N-BOC-L-aspartic acid, each at 4 μg/mL, and diluted in 0.1 formic acid in water (Honeywell, Charlotte, NC, USA). To precipitate the proteins, 80% iced methanol was added, followed by homogenization on the bead beater and incubation for 30 min on ice. Then, the samples were centrifuged at 2000× *g*, 4 °C, for 10 min. The supernatant was transferred to a new tube, followed by a drying process using a nitrogen dryer at 30 °C (Organomation Associates, Inc., Berlin, MA, USA). Then, samples were resuspended with a global metabolomic injection standard solution. The global metabolomic injection standard mix included BOC-L-tyrosine, N-alpha-BOC-L-tryptophan, and BOC-D-phenylalanine (each prepared to 2 μg/mL) diluted in 0.1 formic acid in water. Also, samples were further centrifuged to precipitate any remaining proteins. Finally, the supernatants were transferred to LC vials for analysis. Chemical standards were obtained from Acros Organics (Fairlawn, NJ, USA) unless otherwise stated. Ammonium acetate, and ammonium formate, were obtained from Thermo Fisher Scientific (Waltham, MA, USA).

### 4.3. Analytical Instrumentation

To perform the metabolomics analysis, a Dionex Ultimate 3000 ultra-high-performance liquid chromatography (UHPLC) connected to a Thermo Q exactive Quadrupole-Orbitrap High-Resolution Mass Spectrometer (HRMS) with heated electrospray ionization was utilized (ThermoScientific, San Jose, CA, USA). The analysis process started with reversed-phase liquid chromatography using an Evosphere C18-PFP monodisperse column (100 mm × 2.1 mm, 3.0 μm) (Fortis Technologies Ltd., Cheshire, UK). The separation of metabolites was performed in a gradient elution manner using two mobile phases, including 0.1% formic acid in water (solvent A) and acetonitrile (solvent B). The gradient elution was performed at a flow rate of 0.35 mL/min, as follows: 0–3 min, 100% A isocratic; then, 3–13 min, 0–80% B linear; then, 13–16 min, 80% B isocratic, and 16–16.5 min, 80–0% B linear; and then, finally, 16.5–20 min, 100% A isocratic at 0.6 mL/min to flush the column and achieve equilibration. A 6 µL sample volume was injected for both ionization modes. A complete scan for data acquisition includes positive and negative electrospray ionization at a mass resolution of 35,000, scanning from *m*/*z* 70 to 1000. The specified source parameter settings were a capillary voltage of 3.5 kV, probe temperature of 350 °C, capillary temperature of 320 °C, sheath gas at 40, auxiliary gas at 10, and sweep gas at 1.

### 4.4. Data Processing

The raw data from the mass spectrometry were collected and processed through MZmine 3 [59]. Before starting the data process, RawConverter software 1.1.0.23 was used to convert the raw data file to mzXML format [60]. First, all data processes, including mass detection, peak picking, chromatogram alignment, deconvolution, smoothing, and metabolite identification, were conducted in MZmine. Based on the Metabolomics Standards Initiative guidelines, the metabolites identified in this study were classified as level 1 or 3. To achieve level 1 identification, two orthogonal analytical techniques were applied. Specifically, for level 1, an internal library was used for metabolite identification by matching *m*/*z* within 5 ppm and elution times ±0.2 s in positive mode, and *m*/*z* within 10 ppm and elution times ±0.2 s in negative mode. This internal library was generated using pure analytical standards. For level 3 identification, metabolites were matched based on *m*/*z* values within 5 ppm in positive mode and 10 ppm in negative mode using the Human Metabolome Database. Blank feature filtering was applied to eliminate the background noise by comparing the samples to the extraction blank [61].

### 4.5. Statistical Analysis

The dataset’s statistical analysis and figure generation were performed using Metaboanalyst 6.0, an open-source R-based program [62]. First, the data were normalized to the sum, transformed by log transformation, and then scaled using autoscaling before analysis to calibrate the differences among samples. *p*-values were determined using the two-tailed, unpaired Student’s *t*-test, assuming equal variance, and were adjusted for the false discovery rate. In this report, a *p*-value threshold of ≤0.05 was considered significant.

## Data Availability

Datasets are provided within the article and Supplementary Materials.

## Supplementary Materials

The following supporting information can be downloaded at: https://www.mdpi.com/article/10.3390/ijms26073109/s1.
