# Supplementary material for: Analysis of Metabolomic Reprogramming Induced by Infection with Kaposi’s Sarcoma-Associated Herpesvirus Using Untargeted Metabolomic Profiling
Source: Int J Mol Sci. 2025 Mar 28;26(7):3109. doi: 10.3390/ijms26073109 (PMC11988554; doi:10.3390/ijms26073109)
Supplement: Supplementary file 1 [file ijms-26-03109-s001.zip › List of Supplementary Material.pdf]

Description of Supplementary Materials

Table S1

The Sheet 1 of this file features list includes positive and negative modes, including the identified and non-identified. At the end of each ID, P is positive, and N is negative. Sheet 2 features list includes positive and negative modes, including only identified.

Table S2

The features list includes positive and negative modes, including the identified and non-identified, after applying the measurement of fold change.

Table S3

The features list includes positive and negative modes, including the identified and non-identified, after performing T-test.

Table S4

The list of only significant features includes positive and negative modes, including the identified and non-identified, after performing a volcano plot to measure statistical significance.

Table S5

The list of only significant features includes positive and negative modes, including only identified, after performing a volcano plot to measure statistical significance.

Table S6

The list of upregulated pathways that were affected by using only significant features, including only identified positive and negative modes, after performing pathway enrichment analysis.

Table S7

The list of downregulated pathways that were affected by using only significant features, including only identified positive and negative modes, after performing pathway enrichment analysis.
